# Experimental Energy Consumption of Frame Slotted ALOHA and Distributed Queuing for Data Collection Scenarios

**DOI:** 10.3390/s140813416

**Published:** 2014-07-24

**Authors:** Pere Tuset-Peiro, Francisco Vazquez-Gallego, Jesus Alonso-Zarate, Luis Alonso, Xavier Vilajosana

**Affiliations:** 1 Internet Interdisciplinary Institute (IN3), Universitat Oberta de Catalunya (UOC) C/Roc Boronat 117, Barcelona 08018, Spain; E-Mail: xvilajosana@uoc.edu; 2 M2M Department, Centre Tecnologic de Telecomunicacions de Catalunya (CTTC) Av. Carl Friedrich Gauss 7, Castelldefels 08860, Spain; E-Mails: francisco.vazquez@cttc.es (F.V.-G.); jesus.alonso@cttc.es (J.A.-Z.); 3 Signal Theory and Communications Group, Universitat Politecnica de Catalunya (UPC) Av. Esteve Terradas 7, C4-204, Castelldefels 08860, Spain; E-Mail: luisg@tsc.upc.edu

**Keywords:** energy consumption, data collection, medium access control, frame slotted ALOHA, distributed queuing

## Abstract

Data collection is a key scenario for the Internet of Things because it enables gathering sensor data from distributed nodes that use low-power and long-range wireless technologies to communicate in a single-hop approach. In this kind of scenario, the network is composed of one coordinator that covers a particular area and a large number of nodes, typically hundreds or thousands, that transmit data to the coordinator upon request. Considering this scenario, in this paper we experimentally validate the energy consumption of two Medium Access Control (MAC) protocols, Frame Slotted ALOHA (FSA) and Distributed Queuing (DQ). We model both protocols as a state machine and conduct experiments to measure the average energy consumption in each state and the average number of times that a node has to be in each state in order to transmit a data packet to the coordinator. The results show that FSA is more energy efficient than DQ if the number of nodes is known a priori because the number of slots per frame can be adjusted accordingly. However, in such scenarios the number of nodes cannot be easily anticipated, leading to additional packet collisions and a higher energy consumption due to retransmissions. Contrarily, DQ does not require to know the number of nodes in advance because it is able to efficiently construct an ad hoc network schedule for each collection round. This kind of a schedule ensures that there are no packet collisions during data transmission, thus leading to an energy consumption reduction above 10% compared to FSA.

## 1. Introduction

The Internet of Things (IoT) aims at making domestic, industrial and city-wide processes more efficient and sustainable by revealing real-time information to its stake-holders and enabling them to make informed decisions [1]. Until today, such information has remained hidden to them due to the lack of available infrastructure. However, this is currently changing and Wireless Sensor Networks (WSN) are a key asset because they provide the communications infrastructure that enables to collect data from distributed sensors [2]. In that sense, there is an ongoing paradigm shift in low-power wireless communications towards using single-hop long-range technologies instead of conventional short-range mesh technologies. The rational behind this kind of change is reducing the cost of deploying the communications infrastructure while retaining the capacity of nodes to operate for years using batteries. In practice, this paradigm shift implies that a large number of nodes can now be potentially addressed by each network coordinator, which creates new challenges that need to be investigated and addressed.

In particular, the challenges introduced by single-hop long-range wireless technologies are key in data collection scenarios because the number of nodes present in the network is unknown a priori and is dynamic between consecutive collection rounds. Moreover, since nodes in a data collection scenario only communicate when triggered by the network coordinator, either periodically or on demand, this creates bursty traffic patterns that are a potential source of network congestion and energy expenditure. Given the unknown number of devices and the bursty traffic patterns, as well as the low-power requirements described earlier, designing a low-power Medium Access Control (MAC) protocol is important for data collection scenarios. The MAC layer controls when the radio transceiver receives or transmits and, thus, it determines the average energy consumption of nodes [3]. It is well-known that the energy waste at the MAC layer comes from four sources [4]: idle listening, packet overhearing, packet collisions and protocol overhead. Thus, it is crucial to design MAC protocols that are efficient in these terms.

The MAC layer for data collection scenarios is typically based on Frame-Slotted ALOHA (FSA) due to its simplicity. Other alternatives such as Time Division Multiple Access (TDMA) or Carrier Sense Multiple Access (CSMA) [3] are not used because the number of nodes is unknown a priori or due to the performance effects of the hidden node [5]. In FSA, time is divided into frames which, in turn, are divided into a number of fixed-length slots. Each node to be collected selects one slot of the current frame at random and transmits its data packet. The outcome of each slot can be empty, success or collision. Successful nodes go back to sleep and the process is repeated until all nodes have been successfully collected. Despite its simplicity, it is well-known that the maximum performance of FSA is bounded to around 36.8% due to the effects of contention [6]. Moreover, such efficiency can only be achieved when the number of slots per frame is equal to the number of nodes [7], which is unknown a priori. Over the last decade, different proposals have been made to improve the performance of FSA [8]. These approaches are either based on adapting the number of slots per frame through estimating the number of nodes from collisions or by means of building a query tree prior to collecting the data from nodes.

An alternative to FSA is Distributed Queuing (DQ), which was first presented by Xu *et al.* [9,10] for the distribution of CATV (Cable TeleVision) signals and has later been adapted to wireless communications [11–14]. In short, DQ is a channel access mechanism that ensures collision-free data transmissions and offers a near optimum performance that is independent of the offered traffic and the number of nodes present in the network. DQ evolves from CTA (Collision Tree Algorithm) protocols [15], where the ternary feedback (e.g., empty, collision and success) obtained from the transmission of data packets is used to subsequently split nodes into sub-groups to reduce the collision probability of future data packet transmissions. However, DQ has several advantages over CTA. First, it interleaves the contention resolution process with the transmission of data packets. Second, it uses specific packets that are shorter than data packets to obtain the ternary feedback. Third, it uses the feedback obtained to organize the nodes in two different queues, one to manage collision resolution and the other to manage data transmission. This enables to minimize the effects of contention, thus leading to an increase in network performance and a reduction in the energy consumption.

Taking that into account, in Vazquez *et al.* [16] we proposed an analytic energy consumption model of FSA and DQ for data collection scenarios and validated it using computer simulations. The results showed that in data collection scenarios DQ can reduce the energy consumption by more than 80% compared to FSA thanks to the way it organizes nodes into queues to ensure that there is no contention during data packet transmission. However, an implementation of DQ was not available at that time and, consequently, the results of the energy model were not empirically validated. Thus, the aim of this article is to experimentally validate the energy consumption model of FSA and DQ in a data collection scenario. To do that we build a testbed, implement both FSA and DQ, and conduct a series of experiments to evaluate its energy consumption. The results obtained show that FSA can be very energy efficient when the number of nodes is known in advance and the number of slots per frame adjusted accordingly. However, such conditions are unrealistic and additional energy consumption can be expected. Contrarily, DQ does not require to know the number of nodes in advance, as it is able to dynamically build an ad hoc network schedule to ensure collision free data transmission. On average, DQ offers a 10% reduction in energy consumption compared to the optimal FSA. This adds to the fact that DQ has a MAC layer efficiency close to 100%, whereas FSA can only achieve a MAC layer efficiency around 36.8% [17].

The remainder of the article is organized as follows. Section 2 presents the operation of FSA and DQ. Section 3 presents the analytical energy consumption model of FSA and DQ. Section 4 presents the experiments to validate the energy consumption of FSA and DQ and a discussion of the results obtained. Finally, Section 5 concludes the article.

## 2. Background

This section presents the operation background of Frame-Slotted ALOHA (FSA) and Distributed Queuing (DQ). For a detailed operation of FSA and DQ please refer to [8] and [12] respectively.

### 2.1. Frame Slotted ALOHA

**Figure 1.**
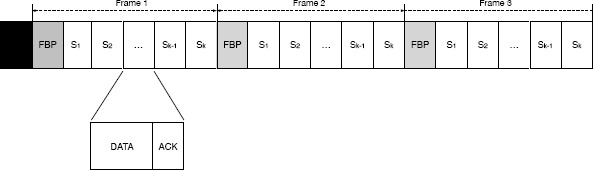
Frame Slotted ALOHA (FSA) time organization. The black square in front of the first frame represents the protocol used to wake-up and synchronize the nodes.

In FSA time is divided into fixed-length frames that repeat over time until data from all nodes has been successfully collected, as depicted in Figure 1. Each frame starts with a feedback period that enables the coordinator to provide information to nodes regarding the number of slots in the current frame, as well as the length of each slot. After the feedback period there are *k* fixed-length data slots that enable nodes to transmit their data packets to the coordinator. Each data slot is divided into two subperiods, data and acknowledgement. The data subperiod enables nodes to transmit its data to the interrogator, whereas the feedback subperiod enables the interrogator to acknowledge the correct reception of the data.

At the beginning of each frame, nodes who have still not been successfully collected by the coordinator select at random one slot of the *k* slots available in the current frame and transmit their data packet. The random number is drawn from a uniform distribution to ensure that all slots have the same probability of being selected. The outcome of each slot can be threefold: empty, collision or success. Empty happens when no node selects that particular slot. Collision happens when two or more nodes select the same slot. Finally, success happens when only one node selects a given slot. Depending on the outcome of each particular slot, the coordinator provides feedback to the nodes. Based on this feedback, each node decides which action to take. Nodes that have been successfully collected go back to sleep until a next collection round begins. Contrarily, nodes of which packets have collided in the current frame wait until the next frame starts and repeat the process. Thus, the data collection process is repeated until the coordinator detects that all the slots of the current frame are empty.

### 2.2. Distributed Queuing

**Figure 2.**
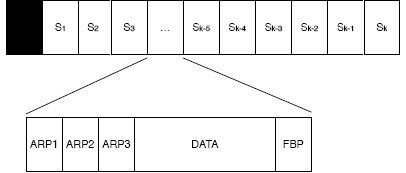
Distributed Queuing (DQ) time organization. The black square in front of the first slots represents the protocol used to wake-up and synchronize the nodes.

In DQ time is divided into fixed-length slots but, contrarily to FSA, the slots are not grouped in frames. As depicted in Figure 2, within each slot three sub-periods are defined, the access request subperiod, the data transmission subperiod and the feedback subperiod. From a node perspective, the data and the feedback subperiods serve the same purpose as in FSA, that is, transmit a data packet to the coordinator and receive a FBP (FeedBack Packet) from the coordinator. However, the access request subperiod serves a different purpose. It enables nodes to request access to the system by transmitting an Access Request Packet (ARP) in one of the *m* available ARP slots. Similarly to FSA, the ARP slot is selected at random, e.g., using a uniform distribution. An ARP is a short packet, compared to a data packet, that enables the coordinator to distinguish between the three states, e.g., empty, collision or success, as described earlier. The number of ARP slots in the access request subperiod can be optimized depending on the number of nodes. A small number of ARP slots increases the network throughput, whereas a large number of ARP slots reduces the time to resolve collisions. However, it has been show in [10] that *m* = 3 is the minimum number that ensures that the system is stable.

Based on the feedback provided by the coordinator in the feedback subperiod nodes are organized into two queues, the Collision Resolution Queue (CRQ) and the Data Transmit Queue (DTQ). On the one hand, the CRQ is used to resolve collisions during the access request subperiod. Nodes that transmit in the same ARP and collide are subsequently grouped together to resolve their collisions in subsequent attempts. This policy works towards creating smaller groups with reduced collision probability at each step which, in the end, ensures a successful ARP transmission no matter the conditions, e.g., number of contending devices. On the other hand, the DTQ is used to queue devices that have successfully transmitted their ARP and are waiting to transmit their data packet to the coordinator. Because only one node can hold each position in the queue, this policy works towards ensuring that no collisions occur during the transmission of data packets. In DQ each queue is represented by two integer numbers, one that is global to the network and the other that is local to each node. The two global integer determine the overall length of each queue, whereas the two local integer determines the current position of the node in each queue.

Finally, there is a set of rules that determine two aspects of the protocol operation. First, how to update the global and local numbers that represent the CRQ and DTQ in each device based on the feedback provided by the coordinator in each slot. Second, what action can each device take in the next slot based on their current position on either of the queues. For example, a node can only hold a position in one or the other queue simultaneously, that is, it can either be waiting to resolve a collision or be waiting to transmit a data packet. Similarly, a node in the CRQ or the DTQ can only transmit if they are at the head of either queue. In contrast to FSA, the data collection process ends when the coordinator notices that both the CRQ and the DTQ are empty, that is, no further nodes are waiting to gain access to the system or have to transmit their data packet.

## 3. Energy Model

The analytic energy consumption model of FSA and DQ for a data collection scenario where a given number of nodes have to transmit a data packet to the coordinator was presented in [16]. However, the analytic model cannot be validated experimentally due to the assumptions made to develop it. Taking that into account, in this section we present a methodology to validate the energy consumption of FSA and DQ experimentally. In both cases the methodology is based on modeling the protocols as a state machine and measuring two parameters. First, the average number of times that a node has to be in each state in order to successfully transmit a data packet to the coordinator. Second, the average energy consumption that a node spends in each state due to radio transceiver activity, e.g., transmit or receive. A similar methodology has been used to evaluate other MAC protocols, such as WirelessHART [18] and IEEE 802.15.4e TSCH (Time Slotted Channel Hopping) [19,20].

### 3.1. Frame Slotted ALOHA

**Figure 3.**
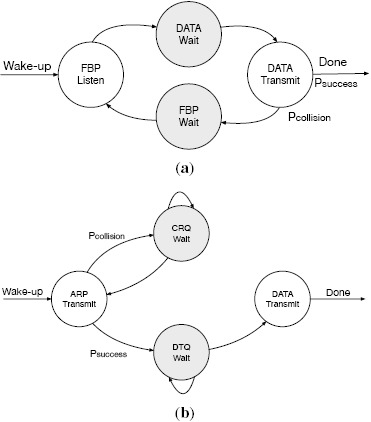
Frame Slotted ALOHA (FSA) and Distributed Queueing (DQ) states. (**a**) Frame Slotted ALOHA (FSA) states; (**b**) Distributed Queuing (DQ) states.

In FSA a node can be in one of the following states: FBP_LISTEN, DATA_WAIT, DATA_-TRANSMIT and FBP_WAIT, as depicted in Figure 3a. The actions that a node has to perform in each of these states are the following:

- FBP_LISTEN: A node is in the FBP_LISTEN at the beginning of each frame. In the FBP_- LISTEN state a node has to receive the FBP from the interrogator. As described earlier, the FBP contains information regarding the number of slots of the current frame and the length of each slot. Based on that information each node independently selects one slot to transmit its data packet. The selection is made at random using a uniform distribution to ensure that all the slots have the same probability of being selected.
- DATA_WAIT: A node is in the DATA_WAIT state while it waits for the selected slot to transmit its data packet. During the DATA_WAIT state the node does not incur in any radio activity.
- DATA_TRANSMIT: A node is in the DATA_TRANSMIT state during the slot selected at random during in the FBP_LISTEN state. In the DATA_TRANSMIT state the node has to transmit its data packet to the coordinator and listen to the acknowledgement. If the transmission is successful the node goes back to sleep immediately. Otherwise, the node moves to the FBP_WAIT state, as described next.
- FBP_WAIT: A node that has collided during the transmission of its data packet enters the FBP_WAIT state, where it waits until the end of the current frame to start the process again by moving to the FBP_LISTEN state. During the FBP_WAIT state the node does not incur in any radio activity.

Given the state machine depicted in Figure 3a, it can be easily seen that in any given frame each node has to exactly be 1 time in the FBP_LISTEN and the DATA_TRANSMIT states and wait *N* − 1 times in the DATA_WAIT and the FBP_WAIT states. Thus, in order to model the average energy consumption of FSA in a data collection scenario it is sufficient to measure the number of times that a node has to be in the DATA_TRANSMIT state, that is, the average number of times that a node has to transmit its data packet for it to be successfully received by the coordinator.

### 3.2. Distributed Queuing

In DQ there are four possible states in which a node can be: ARP_TRANSMIT, CRQ_WAIT, DTQ_WAIT and DATA_TRANSMIT. The actions that a node has to perform in each of these states are the following:

- ARP_TRANSMIT: A node is in the ARP_TRANSMIT state when it transmits an ARP to gain access to the DTQ. In the ARP_TRANSMIT state a node has to transmit an ARP to the coordinator in a randomly selected ARP slot (using a uniform distribution) and receive a FBP from the coordinator in the FBP subsection of the slot.
- CRQ_WAIT: A node is in the CRQ_WAIT state while it is waiting to become the head of the CRQ, which allows it to move back to the ARP_TRANSMIT state and transmit another ARP to gain access to the DTQ. While in the CRQ_WAIT state a node only has to receive a FBP from the coordinator in the FBP subsection of the slot.
- DTQ_WAIT: A node is in the DTQ_WAIT state while it is waiting to become the head of the DTQ, which allows it to move forward to the DATA_TRANSMIT state, as described next. Similarly to the CRQ_WAIT state, in the DTQ_WAIT state a node only has to receive a FBP from the coordinator in the FBP subsection of the slot.
- DATA_TRANSMIT: A node is in the DATA_TRANSMIT state when it becomes the head of the DTQ and is allowed to transmit its data packet. In the DATA_TRANSMIT state a node has to transmit a data packet packet to the coordinator in the DATA subsection of the slot and receive a FBP from the coordinator in the FBP subsection of the slot.

Given the state machine presented in Figure 3b, we can model the average energy consumption of DQ measuring the number of times that a node is in three states: ARP_TRANSMIT, CRQ_WAIT and DTQ_WAIT. We do not need to model the number of times that a node is in the DATA_TRANSMIT state for two reasons. First, because we assume that nodes only have one data packet to transmit. Second, because DQ ensures that there will be no collisions during the transmission of data packets, as only one node that holds the first position in the DTQ is entitled to transmit. In case of collision due to external effects, e.g., interference from another network operating nearby, the node would need to repeat the process again until the data packet is successfully transmitted to the coordinator.

## 4. Energy Consumption Evaluation

Based on the methodology to validate the energy consumption of FSA and DQ presented in the previous section, this section describes the experiments to evaluate the energy consumption of both FSA and DQ, as well as discuss the obtained results. First, we present the research platform and the parameters that have been used to conduct the experiments. Second, we present the methodology followed to conduct the experiments. Third, we present the energy consumption analysis of FSA and DQ. Finally, we discuss the obtained results.

### 4.1. Research Platform

**Figure 4.**
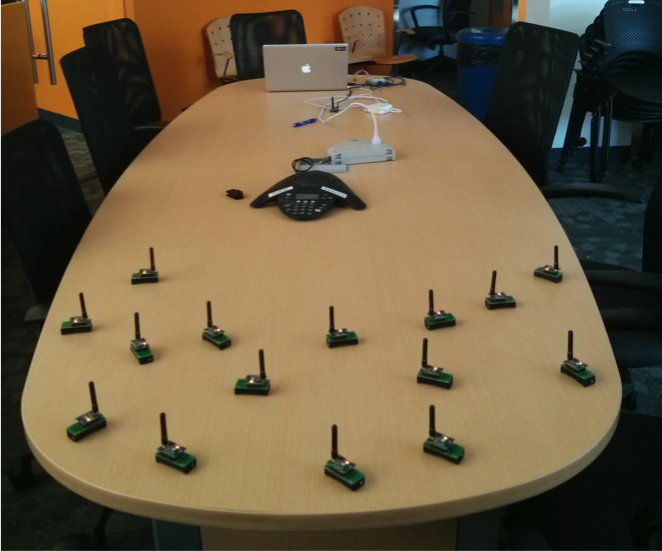
Research platform with 25 nodes, one coordinator and the system manager.

The research platform is composed of 25 nodes and an interrogator connected to a computer that acts as the system manager, as shown in Figure 4. Both the interrogator and the nodes are based on OpenMote-433, a low-power wireless platform build using COTS (Commercial Off-The-Shelf) hardware. Specifically, OpenMote-433 is based on a Texas Instruments CC430 SoC (System on Chip), which embeds an MSP430 16-bit RISC microcontroller, running at 16 MHz with 4 kB of RAM and 32 kB of Flash memory, and a CC1101 radio transceiver, which operates at Sub-GHz bands with data rates of up to 600 kbps and support for amplitude and frequency modulations. In both cases the radio transceiver is tuned to the 433 MHz band using a discrete balun and connected to a *λ*/4 monopole antenna through an SMA connector. Since the experiments are conducted in close range, we have added a 30 dB RF (Radio Frequency) attenuator to the interrogator to ensure that the radio transceiver does not saturate due to the input power. Finally, the nodes are powered using two AAA batteries (3 V, 1500 mAh), whereas the interrogator is powered through the computer USB port.

Table 1 summarizes the physical layer parameters that have been used to conduct the experiments. The parameters used in the experiments are in accordance with the IEEE 802.15.4f amendment [21] to the IEEE 802.15.4 standard [22], which is targeted at active RFID (RadioFrequency IDentification) applications, e.g., data collection scenarios. IEEE 802.15.4f defines three possible data rates, *i.e.*, 31, 100 and 250 kbps, and a type of continuous phase FSK (Frequency Shift Keying) modulation called MSK (Minimum Shift Keying). In our experiments we have used a data rate of 250 kbps, which yields a measured sensitivity of *−*91 dBm for a PER (Packet Error Rate) of 1% transmitting packets of 20 bytes and using a channel bandwidth of 540 kHz [23]. This data rate has been selected because it is equivalent to that of IEEE 802.15.4 and achieves the least energy consumption per bit while offering a range that has been measured to be 1.6 times that of the 2.4 GHz band in real conditions [23].

Table 2 summarizes the energy consumption of the CC1101 radio transceiver within the CC430 SoC. The CC1101 has four possible states: OFF, SLEEP, TRANSMIT and RECEIVE. Each state has a power associated taking into account that the system is supplied at 3 V. We only consider the radio transceiver consumption because the microcontroller is in sleep mode while the radio is transmitting or receiving and is only operating to process the packet.

### 4.2. Research Methodology

To perform the experiments and obtain the results we use the following methodology. By default nodes are in the preamble sampling state [24], where they periodically listen to the channel for a short period of time. When triggered, the interrogator sends a train of wake-up packets to synchronize the nodes present within its communication range. Upon synchronization, nodes enter the data transmit state, in which they transmit a 127 bytes data packet to the interrogator using the appropriate MAC protocol, e.g., FSA and DQ. Once a node has transmitted its data packet, it goes back to the preamble sampling state, where it remains until the next experiment begins. We perform an overall of 6 experiments with *k* = 5, 10, 15, 20, 25, 30 nodes respectively. Each experiment is repeated 100 times to obtain the average, minimum and maximum values.

#### 4.2.1. FSA Experimental Results

To obtain the average energy consumption of FSA according to the model presented in Section 3.1, we determine the number of data packet transmissions that each node has to perform on average. To do so, we conduct an experiment where the nodes participate in a data collection scenario, that is, each node has one data packet to transmit to the coordinator. Because we know the number of nodes (*n*) in the experiment in advance we configure the number of slots per frame (*k*) to the optimal, that is, *k* = *n* [7].

**Figure 5.**
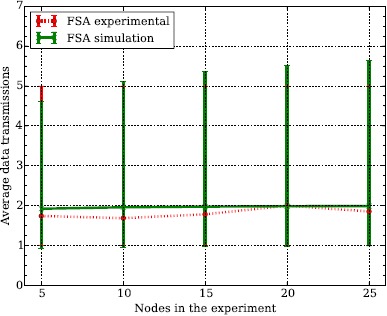
Average number of data packet transmissions using Frame Slotted ALOHA (FSA). Each point is the average of 100 experiments and the error lines are the minimum and maximum values. If the number of slots per frame *k* is equal to the number of nodes *n*, on average data packets need to be retransmitted twice in order to be successful.

The results obtained in the experiments are depicted in Figure 5. On average, each node has to transmit its data packet twice in order for the packet to be successfully received by the coordinator. To validate the experimental results we conduct computer simulations using a Monte Carlo method. In the simulations, each of the *n* nodes selects one of the *k* slots at random to transmit a data packet. The random number follows a uniform distribution to ensure that the probability of selecting any given slot is equal. The simulations for each given *k* are repeated 10, 000 times and the average, maximum and minimum values are computed. As shown in Figure 5, the simulation and experimental results fit, thus validating the experimental results. The differences that can be appreciated between the simulation and experimental results can be explained by the capture effect [25], which is taking place due to the low number of nodes and the FSK modulation scheme that is used to transmit data.

Finally, in FSA the average number of data packet transmissions also determines the number of FBP_LISTEN, DATA_WAIT and FBP_WAIT states. For a given number of nodes *n*, the optimal number of slots per frame is *k* = *n*. Thus, if a given node has to transmit its data packet twice, it will also need to listen to two FBP, as well as remain 2*k* − 2 slots waiting either prior to transmitting the data packet or waiting until the following FBP.

#### 4.2.2. DQ Experimental Results

To obtain the average energy consumption of DQ according to the model presented in Section 3.2, we determine two things. First, the average number of ARPs that a node has to transmit in order to request access to the system. Second, the average number of slots in which a node has to wait in a queue, either the CRQ or the DTQ.

**Figure 6.**
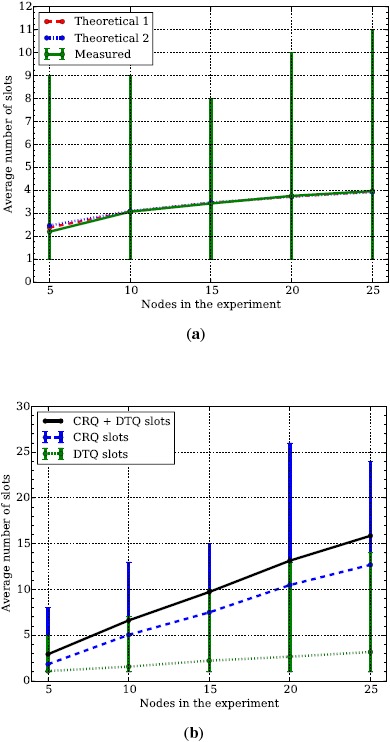
Average number of ARP and CRQ + DTQ in required to be able to transmit a data packet using Distributed Queuing (DQ). Each point is the average of 100 experiments and the error lines are the minimum and maximum values. The average number of ARP increases logarithmically, whereas the average number of CRQ + DTQ increases linearly. (**a**) ARP count; (**b**) CRQ + DTQ count.

First, we validate the average number of ARP that a node needs to transmit in order to gain access to the DTQ. According to [15,16], the number of slots in which a node tries to access the DTQ can be approximated by $d_{N}={\text{log}}_{m}(n-1)+\left(\frac{1}{2}+\frac{\gamma }{\text{log}(m)}\right)+\frac{1}{2n\text{log}(m)}$, where *γ* = 0.5772 (Euler constant). As depicted in Figure 6a, the measured data fits the theoretical data perfectly. For *m* = 3 the theoretical model can be simplified to *d_n_* = *log*_3_(*n* − 1) + 1, which corresponds to the average number of levels in the ternary tree plus the root.

Second, we validate the average number of slots that a node has to wait in the CRQ queue in order to transmit an ARP to join the DTQ, as well as the average number of slots that a node has to wait in the DTQ queue in order to reach its head and be able to transmit the data packet. As depicted in Figure 6b, the number of slots waiting in the CRQ and the DTQ queues is linearly proportional to the number of nodes present in the experiment. This kind of result is expected because either a priori or a posteriori nodes have to wait in a queue which average size will depend on the number of nodes within the experiment.

### 4.3. Energy Consumption Analysis

The average number of states that a given node of the network has to go through in order to transmit its data packet to the coordinator in a data collection round using FSA and DQ, as obtained in Section 4.2.1 and Section 4.2.2 is summarized in Table 3.

**Figure 7.**
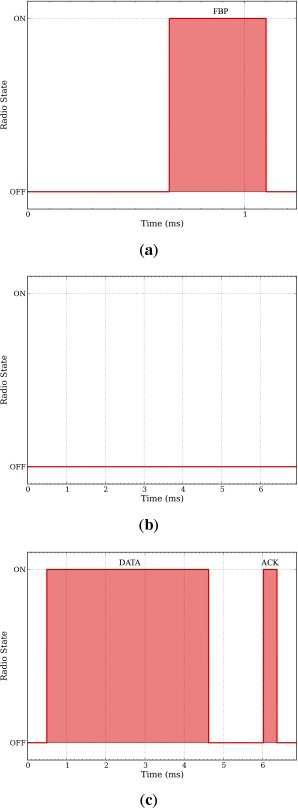
Frame Slotted ALOHA (FSA) states and radio activity. The total slot length is 6.85 ms, yielding an overall of 145 packets per second. In FSA the FBP_LISTEN state duration is shorter (1.25 ms) than other states. (**a**) FBP_LISTEN state. OFF = 800 *μ*s, RX = 450 *μ*s; (**b**) FBP_WAIT and DATA_WAIT states. OFF = 6.85 ms; (**c**) DATA_TRANSMIT state. OFF = 2.3 ms, RX = 450 *μ*s, TX = 4.1 ms.

**Figure 8.**
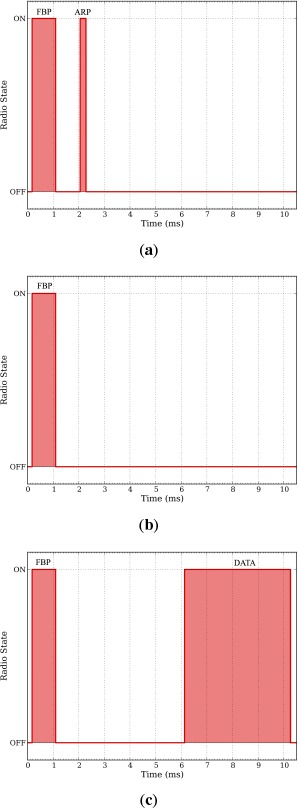
Distributed Queuing (DQ) states and radio activity. The total slot length is 11.9 ms, yielding an overall of 84 packets per second. In DQ the duration of all the states is the same (11.9 ms). (**a**) ARP_TRANSMIT, OFF = 10.6 ms, RX = 1 ms, TX = 300 *μ*s; (**b**) CRQ_WAIT and DTQ_WAIT. OFF = 10.9 ms, RX = 1 ms; (**c**) DATA_TRANSMIT. OFF = 6.8 *μ*s, RX = 1 ms, TX = 4.1 ms.

Measuring the average energy consumption in each of these states, it is now possible to obtain the average energy consumption of a given node. To do so, we first measure the time spent in each state for FSA and DQ using a logic analyzer. The timing is captured toggling on and off a pin when the radio enters or exits the transmit and receives modes. As stated in Section 3, in FSA a node can be in four different states: FBP_LISTEN, DATA_WAIT, DATA_TRANSMIT and FBP_WAIT. Conceptually, the DATA_WAIT and FBP_WAIT states are the same because the node remains waiting in sleep mode, e.g., no radio activity. Contrarily, the FBP_LISTEN and the DATA_TRANSMIT states are different, as a node has to execute different actions, e.g., receive a FBP or transmit a DATA packet plus receive an ACK packet. Similarly, in DQ a node also can be in four states: ARP_TRANSMIT, CRQ_WAIT, DTQ_WAIT and DATA_TRANSMIT. Here, the CRQ_WAIT and the DTQ_WAIT states are the same, e.g., the node remains waiting and only needs to receive a FBP. Contrarily, the ARP_TRANSMIT and the DATA_TRANSMIT are different. In the ARP_TRANSMIT state the node has to transmit an ARP packet to request access to the system and receive a FBP from the coordinator. In the DATA_TRANSMIT state the node has to transmit a DATA packet and receive a FBP from the coordinator. The timing of the radio in each state using FSA and DQ is depicted in Figures 7 and 8 respectively.

Based on the timing and the radio activity in each of these states, as depicted in Figures 7 and 8, and the power consumption in each radio state, summarized in Table 2, we can now obtain the average energy spent in each state for both FSA and DQ. Table 4 summarizes the energy consumption of FSA and DQ in each state.

Finally, with the energy spent in each state and the average number of times that a node has to be in each state in order to transmit a data packet, as summarized in Table 3, it is possible to calculate the average energy consumption of both FSA and DQ. We do so by multiplying the average number of times that a node is in each state by the energy consumption in each of these states depending on the number of nodes. The results are summarized in Table 5.

### 4.4. Discussion

According to Table 5, FSA achieves a lower energy consumption than DQ regardless of the number of nodes in the network. However, the results obtained for FSA are the optimal case, that is, when the coordinator knows the number of nodes (*n*) in the network a priori and, thus, it is able to adjust the number of slots per frame (*k*) to the optimal case, e.g., *k* = *n*. Such an assumption is not realistic in data collection scenarios because the number of nodes may change at each data collection period. In case the coordinator does not have such information in advance, an impact on the performance and energy consumption of FSA can be expected. On the one hand, if the number of slots per frame is larger than the number of nodes, e.g., *k* > *n*, the data collection time will be affected because a large number of slots will remain empty. On the other hand, if the number of slots per frame is smaller than the number of nodes, e.g., *k* < *n*, a greater energy consumption can be expected because the collision probability will be higher and, thus, the nodes will have to transmit their data packet additional times. In contrast, in DQ the coordinator does not need to know the number of nodes a priori because the collision resolution mechanism and the distributed queues work towards creating an ad hoc network schedule that ensures collision free transmission of data packets. In addition, the collision resolution process is interleaved with data transmission, thus improving the data collection delay compared to other protocols.

**Figure 9.**
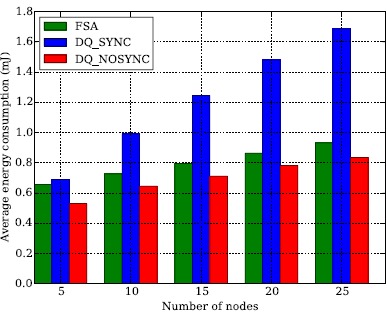
Energy consumption of Frame Slotted ALOHA (FSA) and Distributed Queuing (DQ) with both synchronization and no synchronization. Reducing the number of FBP required to maintain synchronization leads to a reduction in energy consumption that makes DQ more energy efficient and easier to implement than FSA.

Another downside of FSA comes from the implementation point of view. In FSA there is no mechanism to recover the clock synchronization for the duration of a frame. Crystals clocks running at 32.768 Hz are typically used as time references to ensure proper protocol operation, e.g., at which slot should a node wake up and transmit the data packet to the coordinator. However, crystal clocks are not perfect and drift with respect to each other depending on many factors, e.g., aging and temperature. For example, two crystals that are rated at 20 ppm, a typical value, can drift as much as 40 ppm or 40 *μ*s per second with respect to each other, one going *fast* and the other going *slow*. Considering a guard interval of 16 clock ticks (488.281 *μ*s) between two consecutive slots, a node will be out of synchronization after 12 s. Thus, considering the current time length of a slot (6.85 ms), the maximum length of a frame is limited to 1750 slots. Despite there are temperature compensated crystals (2 ppm), drift poses a limitation to the number of nodes that a network can support using FSA. In contrast, in DQ the FBP packet can be used as a mechanism to maintain synchronization, e.g., ensure that clock drift does not lead to packet collisions because a node transmits out of its bounds. In that sense, it is worth noting that the current implementation of DQ listens to all FBP while waiting in the CRQ and the DTQ queues in order to maintain synchronization. However, from an implementation perspective it is possible to only listen to enough FBP to maintain clock synchronization within bounds. In such conditions the number of wait states in the CRQ and DTQ would be the same, but the energy consumption in such states would drop from 65.2 *μ*J to 10.9 *μ*J. Such a reduction in the CRQ and DTQ wait states would lead to important savings in the overall energy consumption, as summarized in Table 6 and depicted in Figure 9.

Finally, there are two other optimizations that can be introduced to further improve the performance and energy consumption of DQ with respect to FSA. First, it is possible to reduce the number of bytes devoted to the ARP. Currently ARP packets are 13 bytes long because it includes a radio preamble (4 bytes), synchronization word (4 bytes), payload length (1 byte), node address (2 bytes) and the payload checksum (2 bytes). Such information is included because the mechanism to decide the outcome of an ARP, e.g., empty, success or collisions, is based on the node address and the payload checksum. However, this number could be reduced implementing an advanced collision detection mechanism, e.g., using signal processing techniques. Second, it is possible to increase the number of ARP slots in the access subperiod of a slot. Currently, we use *m* = 3 because it is the minimum that ensures a stable system [10]. However, a larger *m* would lead to a faster collision resolution and, thus, lower energy consumption because nodes would need to transmit a lower number of ARPs to gain access to the DTQ.

## 5. Conclusions

This paper has empirically evaluated the energy consumption of two MAC protocols, Frame Slotted ALOHA (FSA) and Distributed Queuing (DQ), for data collection scenarios in smart cities. The results show that in the optimal case, that is, when the number of slots per frame is equal to the number of nodes present in the network, FSA consumes less energy than DQ. However, the optimal case for FSA is difficult to achieve in real scenarios for two reasons. First, the number of nodes is unknown a priori by the coordinator. Second, the number of nodes may change in each data collection period, thus requiring additional algorithms to adapt the number of slots per frame. Contrarily, DQ does not require to know the number of nodes in advance, yet it is capable of providing an energy consumption that is more than 10% lower than FSA if it only listens to enough FBP to maintain clock synchronization within bounds. Taking that into account, this paper concludes that DQ is an interesting alternative for data collection scenarios where the traffic is bursty and the number of nodes is dynamic.

## Figures and Tables

**Table 1.** Physical layer parameters according to the IEEE 802.15.4f standard.

**Parameter**	**Value**
Frequency band	433 MHz
Channel number	2
Channel frequency	433.380 MHz
Channel bandwidth	540 kHz
Modulation scheme	MSK
Data rate	250 kbps
Transmit power	0 dBm
Sensitivity	−91 dBm

**Table 2.** Power consumption of the CC430 System on Chip (SoC) in the different states. The differences in OFF, SLEEP and RECEIVE are due to the static consumption of other onboard peripherals, e.g., temperature, humidity and acceleration sensor. The difference in TRANSMIT is due to a higher transmit power to compensate for additional RF losses, e.g., balun.

**States**	**Data Sheet (mW)**	**Measured (mW)**
OFF	0.3	1
SLEEP	5.1	5.67
TRANSMIT	50.4	60.9
RECEIVE	54	54.3

**Table 3.** Average number of states for Frame Slotted ALOHA (FSA) and Distributed Queuing (DQ) that a node has to be in to transmit a data packet to the coordinator depending on the number of nodes in the network.

		**Nodes**				
	**States**	**5**	**10**	**15**	**20**	**25**
	FBP_LISTEN	2.0	2.0	2.0	2.0	2.0
**FSA**	FBP_WAIT, DATA_WAIT	8.0	18.0	28.0	38.0	48.0
	DATA_TRANSMIT	2.0	2.0	2.0	2.0	2.0

	ARP_TRANSMIT	2.2	3.1	3.4	3.8	4.0
**DQ**	CRQ_WAIT, DTQ_WAIT	3.0	6.5	10.0	13.1	16.0
	DATA_TRANSMIT	1.0	1.0	1.0	1.0	1.0

**Table 4.** Average energy consumption in each of the Frame Slotted ALOHA (FSA) and Distributed Queuing (DQ) states. The energy consumption in each DQ state is larger than in FSA due to listening the FBP in each slot.

**Protocol**	**State**	**Energy Consumption**
	FBP_LISTEN	25.235 *μ*J
**FSA**	DATA_WAIT, FBP_WAIT	6.850 *μ*J
	DATA_TRANSMIT	276.425 *μ*J

	ARP_TRANSMIT	83.170 *μ*J
**DQ**	CRQ_WAIT,DTQ_WAIT	65.200 *μ*J
	DATA_TRANSMIT	310.900 *μ*J

**Table 5.** Energy consumption of Frame Slotted ALOHA (FSA) and Distributed Queuing (DQ). The FSA/DQ quotient represents the energy savings of using FSA with respect to DQ. In this case, using the optimal FSA leads to a reduction in energy consumption between 5% to 45% compared to DQ.

	**Number of Nodes**				

**Protocol**	**5**	**10**	**15**	**20**	**25**
**FSA**	0.658 mJ	0.726 mJ	0.795 mJ	0.863 mJ	0.932 mJ
**DQ**	0.689 mJ	0.992 mJ	1.245 mJ	1.481 mJ	1.686 mJ
**FSA/DQ**	95.5%	73.2%	63.8%	58.3%	55.3%

**Table 6.** Energy consumption of Frame Slotted ALOHA (FSA) and Distributed Queuing (DQ) with no synchronization. The FSA/DQ quotient represents the additional energy expenditure of using FSA with respect to DQ. Assuming perfect synchronization, the optimal FSA has an additional energy that is between 10% and 24% higher than DQ.

	**Number of Nodes**				

**Protocol**	**5**	**10**	**15**	**20**	**25M**
**FSA**	0.658 mJ	0.726 mJ	0.795 mJ	0.863 mJ	0.932 mJ
**DQ**	0.529 mJ	0.646 mJ	0.713 mJ	0.783 mJ	0.834 mJ
**FSA/DQ**	24.3%	12.5%	11.6%	10.3%	11.8%
